# Defense mechanisms in individuals with depressive and anxiety symptoms: a network analysis

**DOI:** 10.3389/fpsyg.2024.1465164

**Published:** 2024-11-08

**Authors:** Mariagrazia Di Giuseppe, Gabriele Lo Buglio, Erika Cerasti, Tommaso Boldrini, Ciro Conversano, Vittorio Lingiardi, Annalisa Tanzilli

**Affiliations:** ^1^Department of History, Humanities and Society, University of Rome Tor Vergata, Rome, Italy; ^2^Department of Dynamic and Clinical Psychology, and Health Studies, Faculty of Medicine and Psychology, Sapienza University of Rome, Rome, Italy; ^3^Department of Psychology and Educational Science, Pegaso Telematic University, Naples, Italy; ^4^Department of Surgical, Medical and Molecular Pathology, Critical and Care Medicine, University of Pisa, Pisa, Italy

**Keywords:** defense mechanisms, DMRS-SR-30, depression, anxiety, network analysis, COVID-19

## Abstract

**Background:**

Defense mechanisms play a crucial role in depression and anxiety. The current study aimed at estimating the network structure of defense mechanisms in individuals with symptoms of depression and anxiety to understand the most central defenses and relevant connections. Moreover, we aimed at examining the associations between defense mechanisms and symptoms.

**Methods:**

We employed the Symptom Checklist-90 to recruit 655 individuals with depressive and anxiety symptoms during the first wave of the COVID-2019 Pandemic in Italy. Defense mechanisms were assessed with the DMRS-SR-30.

**Results:**

Results showed a main component in the network graph featuring 16 defense mechanisms. Self-assertion was the most central node in the network, displaying positive and negative connections with an array of mature and immature defenses, respectively. Among immature defenses, passive aggression was the most interconnected node. Some mature defenses (i.e., humor, affiliation, and sublimation) were not connected to other nodes. A range of defense mechanisms were associated with anxiety and depressive symptoms.

**Conclusions:**

This is the first research effort supporting the conceptualization of defense mechanisms as a complex system. Results suggest that defense mechanisms of the same cluster (e.g., mature defenses) play different roles in the network. Central defenses (i.e., self-assertion and passive aggression) detected in this study may be promising intervention targets.

## Introduction

According to the main theoretical conceptualizations, defense mechanisms (or, simply, defenses) are pivotal concepts to understand psychological functioning and human development (Vaillant, 2000; McWilliams and Weinberger, 2003; Cramer, 2015; Di Giuseppe and Lingiardi, 2023). Defenses operate mostly, but not exclusively, out of consciousness (Perry, 1990), mediating the relationship between emotional conflicts and external stressors (Perry, 2014). Fueled by seminal conceptualizations of Freud (1894), a variety of theoretical contributions and research efforts have been generated on this topic, leading to well-established models assisting therapists and researchers in the identification of defense mechanisms in clinical and research settings. Particularly, the hierarchical model, proposed by Vaillant (1971, 1977) and operationalized by Perry in the *Defense Mechanisms Rating Scales* (DMRS; Perry, 1990), collocates defenses in a continuum between the maturity and the immaturity pole; in this model, most mature defenses are associated with adaptive responses and high awareness and resilience, while most immature defenses are associated with maladaptive responses, low awareness and psychological distress (Perry et al., 2022; Rice and Hoffman, 2014; Tanzilli et al., 2022; Békés et al., 2023; Carone et al., 2023; Martino et al., 2023; Messina et al., 2023).

The DMRS hierarchy describes 30 defense mechanisms organized into seven defense levels, each of which has a specific defensive function that protects the individual from anxiety, or a sense of threat from internal or external sources, or conflicts (Perry, 2014). From the least to the most adaptive defense levels are: (1) *Action defense level*, including defenses as acting out, passive aggression, and help-rejecting complaining; (2) *Major image-distorting defense level*, including defenses as splitting of self-image, splitting of object's image, and projective identification; (3) *Disavowal defense level*, including defenses as denial, rationalization, projection, and autistic fantasy; (4) *Minor image-distorting defense level*, including defenses as idealization of self and others' image, devaluation of self and others' image, and omnipotence; (5) *Neurotic defense level*, including defenses as repression, dissociation, reaction formation, and displacement; (6) *Obsessional defense level*, including defenses as isolation of affects, intellectualization, and undoing; and (7) *High-adaptive defense level*, including defenses as affiliation, altruism, anticipation, humor, self-assertion, self-observation, sublimation, and suppression. All DMRS measures, including the one applied to this study, refer to the hierarchical model (Di Giuseppe, 2024). Furthermore, defense levels can be organized into three defensive categories of maturity, namely mature, neurotic, and immature. The immature defensive category is the least adaptive and includes defenses belonging to levels 1 to 4. The neurotic defensive category is in the middle of the hierarchy and includes defenses belonging to levels 5 and 6. Finally, the mature defensive category is on the top of the hierarchy and includes defenses belonging to level 7. Since its deep, comprehensive and empirical-based conceptualization of defenses, the DMRS has inspired the inclusion of specific axis for defense mechanisms assessment in widely used diagnostic manuals (American Psychiatric Association, 1994; Lingiardi and McWilliams, 2017) and it is nowadays known as the closest to a gold-standard method for studying defenses (Silverman and Aafjes-van Doorn, 2023).

Several studies have highlighted the association between specific defense mechanisms and depression (Høglend and Perry, 1998; DeFife and Hilsenroth, 2005; Olson et al., 2009; Martino et al., 2020; Fiorentino et al., 2024) and anxiety (Olson et al., 2009). For example, Fiorentino et al. (2024) underscored the over-reliance on non-mature defenses (i.e., neurotic and immature defenses) in depressive individuals. Moreover, Olson et al. (2009) highlighted that panic disorder is characterized by dissociation.

Notably, depression and anxiety represent the most widespread mental health concerns and often co-occur (Galli et al., 2019; Boldrini et al., 2020; World Health Organization, 2022; Ierardi et al., 2023; König et al., 2023). Such a co-occurrence phenomenon has been referred to as comorbidity (Gelo et al., 2015a,b; Lenzo et al., 2020; Nordgaard et al., 2023; Lo Buglio et al., 2024), a concept that may be considered at least partially artifactual (Borsboom and Cramer, 2013; Vita et al., 2020; Borsboom et al., 2021; Martino et al., 2021). Even though the interconnections between symptoms of comorbid mental disorders have been the focus of several studies (Gelo and Manzo, 2015; Conversano et al., 2023; Boldrini et al., 2024), little knowledge is available on the complex interplay of defense mechanisms in individuals with both depressive and anxiety symptoms.

With the aim of advancing the knowledge on the complex interactions among variables (Gelo et al., 2012, 2015b; Casula et al., 2023; Klocek and Riháček, 2023; Parolin et al., 2023; Sergi et al., 2023), promising findings have been generated within the so-called “network approaches” focusing on associations between pairs of variables in the data, while conditioning on all the other variables (i.e., partial correlations, see “network analysis” section for details) (Borsboom, 2017; Borsboom et al., 2021). Recent advancements in the field provide statistical tools useful for the interpretation of both network properties (e.g., most central variables) and relevant links between them (Borsboom et al., 2021). As an example of a complex system, we can consider a flock of birds, in which the behavior of the former is the result (an “emerging property”) of the interplay of the latter (Borsboom et al., 2022). Similarly, defensive functioning originating from the complex interplay of defense mechanisms may be mirrored and represented by a network structure originated from the complex interconnections of defense mechanisms operating all together. However, even though complexity is (mostly) an implicit assumption of theories and models on defense mechanisms, complex models on defenses are still in their infancy. Research within the network approach may provide promising tools for the transdiagnostic assessment of individuals with depressive and anxiety symptoms, informing the identification of consistent intervention targets. Moreover, it could advance knowledge by explicitly framing defenses as complex systems in individuals with comorbid mental health conditions.

### Aim

Given these premises, the current study aimed at investigating the defensive functioning of individuals with high levels of symptoms of depression and anxiety as a network structure, identifying defenses playing a relevant role in the network. Moreover, we also aimed at investigating the associations between defense mechanisms and depressive and anxiety symptoms.

## Methods

### Participants

Participants were extracted from a larger sample of 6,412 adult responders to an online survey launched to test the psychological impact of COVID-19 during the first wave of the pandemic. Previous studies have investigated participants' responses focusing on several socio-demographic and psychological aspects (Di Giuseppe et al., 2022). For the purpose of this study, we selected about 10% of the participants who self-reported to have high depression and anxiety symptoms at the time they responded to the survey (see Procedures). Selected responders were 655 in total, mostly female adults (*N* = 392; 59.8 %), while about 19.7% (*N* = 129) were male and 27.2% (*N* = 178) were either male or female younger than 30 years of age.

### Measures

To evaluate defense mechanisms and symptoms of depression and anxiety we applied the Italian version of two well-validated self-report questionnaires as the DMRS-SR-30 and the SCL-90.

The *Defense Mechanisms Rating Scales*-*Self-Report-30* (DMRS-SR-30; Di Giuseppe et al., 2020) is a 30-item questionnaire developed from the gold-standard DMRS theory (Perry, 1990, 2014) to self-assess the whole hierarchy of defense mechanisms (Di Giuseppe and Perry, 2021). The DMRS-SR-30 provides scores for the overall defensive functioning (ODF), three factors of defensive maturity, seven hierarchically ordered defense levels and 28 constituent defenses. Both the English and Italian version of the measure showed strong psychometric properties (Di Giuseppe et al., 2020; Prout et al., 2022), mostly replicated in the German (Volkert et al., 2022) and Turkish (Yilmaz et al., 2024) version of the scale.

The *Symptoms Checklist-90* (SCL-90; Derogatis and Cleary, 1977) is a 90-item questionnaire developed to measure psychological symptoms and distress. It is designed to be appropriate for use with individuals from the community, as well as individuals with either medical or psychiatric conditions (Gomez et al., 2021). The SCL-90 involves nine primary symptom dimensions (i.e., somatization, obsessive-compulsive, interpersonal sensitivity, depression, anxiety, hostility, phobic anxiety, paranoid ideation, and psychoticism), but in this study we considered only depression (DEP) and anxiety (ANX) subscales. Because it is one of the most comprehensive and widely used scales addressing psychopathological symptoms, the SCL-90 has been largely validated in several languages and populations, including Italian (Cassano et al., 1999; Prunas et al., 2012).

### Procedures

Data were collected from March 13 to April 6, 2020, during the first lockdown imposed by the Italian Government to contrast COVID-19 pandemic spreading. Participants were recruited via social media using snowball sampling and they were asked to give their approval on personal data treatment for research purposes. All procedures followed the ethical standards and were approved by the Ethics Committee of the Local Institution.

Participant selection was made following the criteria published in reference to the Italian version of the Symptom Checklist-90-Revised (SCL-90-R; Sarno et al., 2011). From the best of our acknowledge, this is the only published documents indicating cut-off scores for single SCL subscales (i.e., low, moderate, and high level of symptoms), further differentiated by age (i.e. below vs. above 30 year of age) and gender (i.e., male vs. female). According to Sarno and colleagues' guidelines for determining high levels of depression and anxiety using the SCL-90-R (Sarno et al., 2011), Table 1 shows mean, standard deviation, and cut-off level for depression and anxiety among four subgroups of males and females. We included only individuals scoring above the cut-off levels that indicate the most severe symptoms of both anxiety and depression.

**Table 1:** Means, standard deviations and cut-offs for depression and anxiety.

	**SCL-90 depression (DEP)**			**SCL-90 anxiety (ANX)**		
	***Mean***	***SD***	***Cut-off***	***Mean***	***SD***	***Cut-off***
**Age**<**30 years**						
Males (*N =* 44)	2.34	0.45	1.62	1.99	0.47	1.40
Females (*N =* 134)	2.67	0.40	2.00	2.47	0.45	1.80
**Age** > **30 years**						
Males (*N =* 85)	2.47	0.55	1.08	2.06	0.78	1.00
Females (*N =* 392)	2.36	0.46	1.62	2.17	0.50	1.40

### Network analysis and correlations

A network approach was applied to investigate the interaction between different defense mechanisms. In network modeling, “node” refers to each variable included in the network structure while “edge” denotes a link between two nodes, representing the presence of a conditional dependence between the corresponding variables (dependence between two variables while controlling for all the other variables; Borsboom et al., 2021). If two nodes in the network are not connected through an edge, the corresponding variables are conditionally independent. Edges are estimated from partial correlations which, in turn, are computed from measured correlations (i.e., the correlation matrix). In this study all the defense mechanisms scores are evaluated with the DMRS-SR-30 as variables for the estimation of an undirected and weighted network. Black and red edges indicate positive and negative connections, respectively—-the ticker the edge, the stronger the connection. We used a Gaussian Graphical Model (GGM) and adopted the *EBICglasso* estimator, which shrinks to zero weak associations (dropped edges are not relevant to explain the data covariation structure). To examine the centrality of each defense mechanism in the network, we computed the following indices of centrality: (a) strength (i.e., the sum of the absolute edge weights for each node), closeness (i.e., the inverse of the sum of the distances of the target node from all remaining nodes) and betweenness (i.e., the number of shortest paths between any two nodes that pass through a specific node) (Opsahl et al., 2010; Costantini et al., 2015; Borsboom et al., 2021). To assess the robustness of the network structure, we estimated the correlation stability coefficient (CS; the maximum proportion of the sample that can be dropped with recalculated indices that correlate at least 0.7 with the indices of the whole original sample). A value of 0.25 is considered acceptable and a value of 0.50 is recommended by current methodological guidelines. Confidence intervals were computed with *bootstrapping* (number of boots = 2,500) to examine the variability of edge-weights. The network analysis was conducted in line with relevant instructions in literature (Costantini et al., 2015; Borsboom, 2017; Epskamp and Fried, 2018). We employed the packages *qgraph* and *bootnet* of the statistical program R (version 4.2.2; R Core Team, 2016).

Finally, we employed SPSS version 27.0 (IBM Corp, 2020) to compute the Pearson correlations between all DMRS-SR-30 subscales and the two SCL-90 subscales for depression and anxiety.

## Results

### Network of defense mechanisms

**Figure 1:**
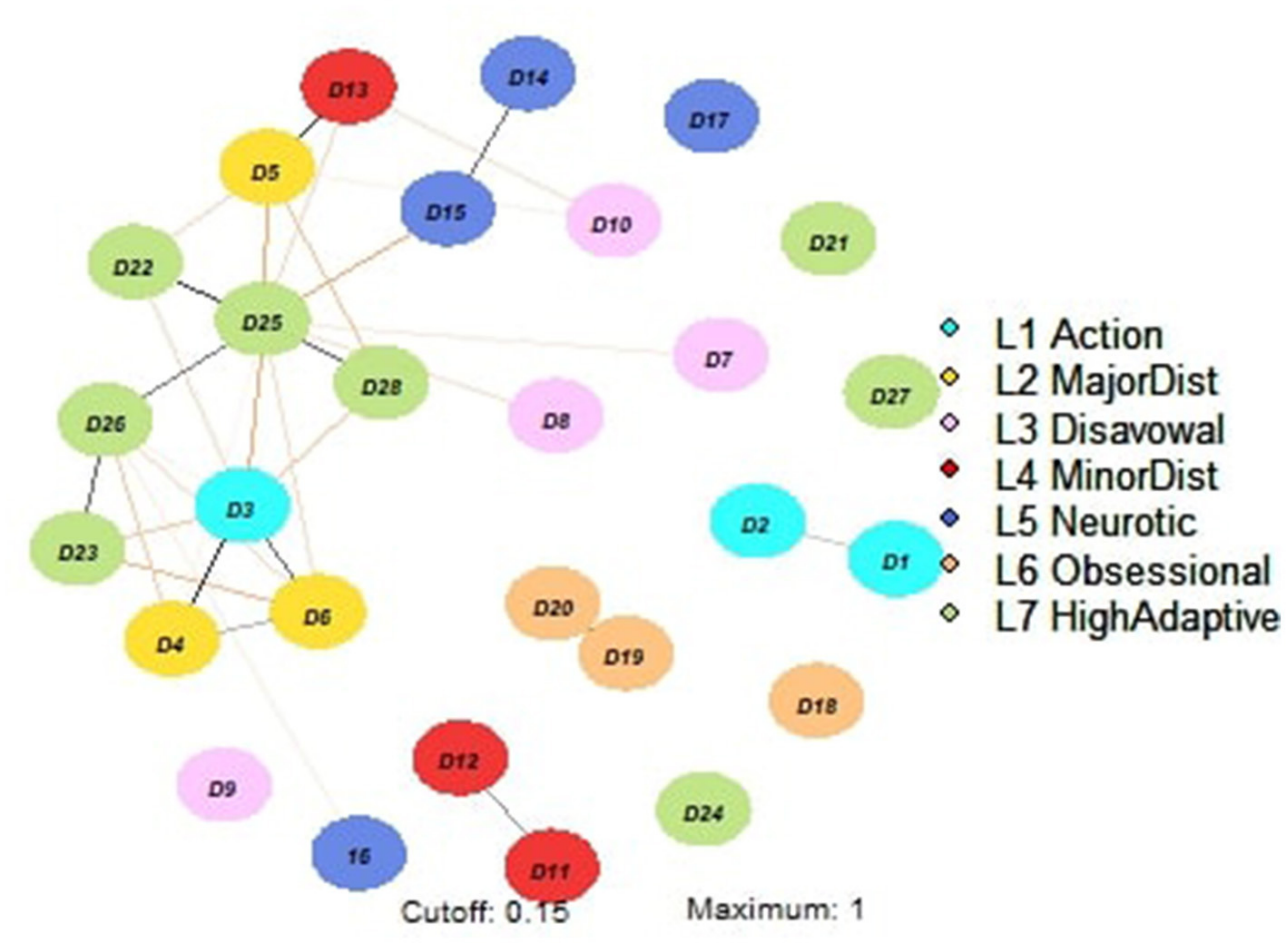
Defense Network of individual with high level of depression and anxiety. Node colors refer to a priori symptom domains (see legend), and numbers refer to specific individual items (i.e., defense mechanisms). The associations are either positive (colored black) or negative (colored red), with thicker lines representing stronger associations. D1 Acting out, D2 Help-rejecting complaining, D3 Passive aggression, D4 Splitting of other's image, D5 Splitting of self-image, D6 Projective identification, D7 Autistic fantasy, D8 Projection, D9 Rationalization, D10 Denial, D11 Omnipotence, D12 Idealization, D13 Devaluation, D14 Repression, D15 Dissociation, 16 Reaction formation, D17 Displacement, D18 Undoing, D19 Intellectualization, D20 Isolation of affect, D21 Affiliation, D22 Altruism, D23 Anticipation, D24 Humor, D25 Self-assertion, D26 Self-observation, D27 Sublimation, and D28 Suppression.

**Figure 2:**
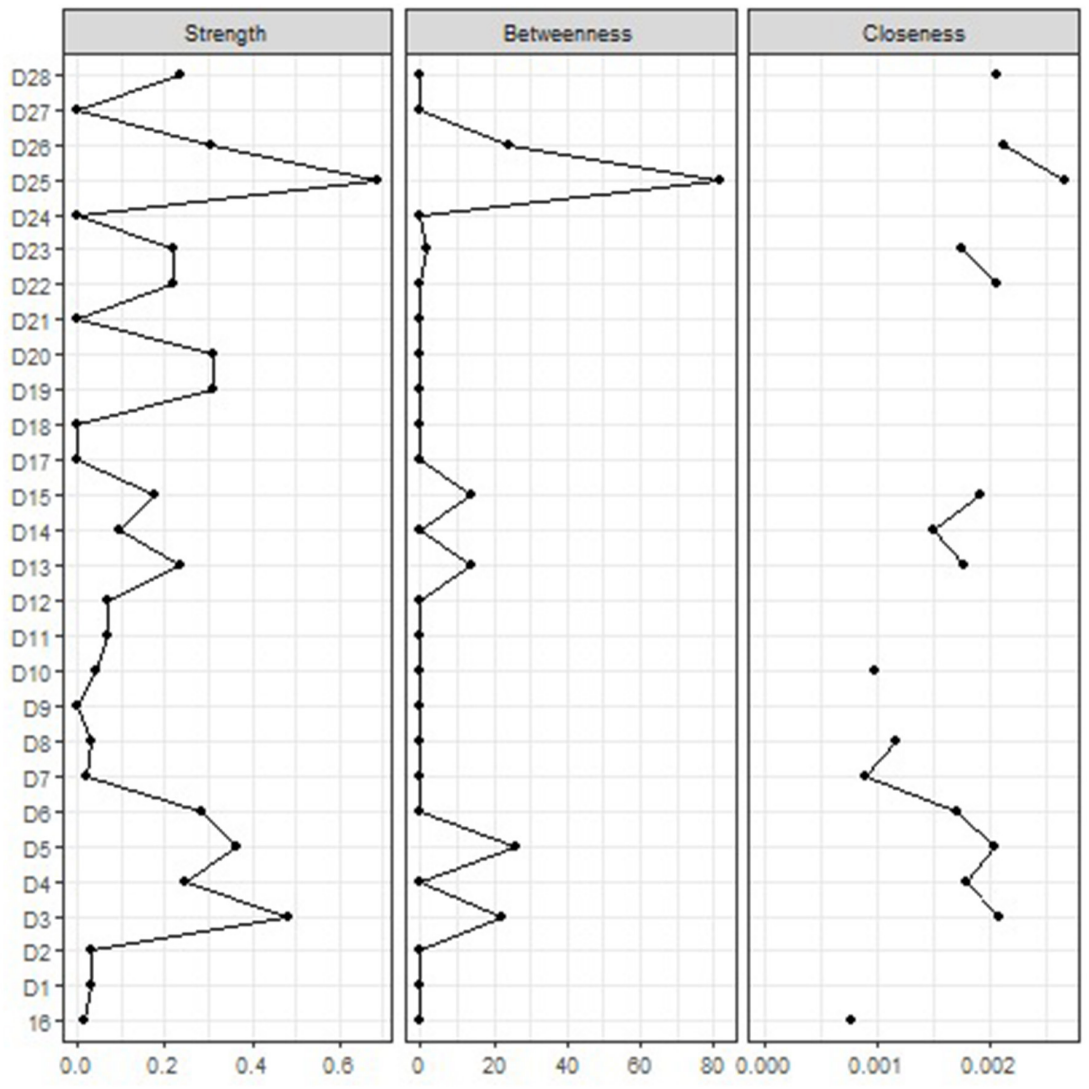
Centrality indices. Centrality indices (i.e., node strength, closeness and betweenness) are shown as standardized *z*-scores. Legend. D1 Acting out, D2 Help-rejecting complaining, D3 Passive aggression, D4 Splitting of other's image, D5 Splitting of self-image, D6 Projective identification, D7 Autistic fantasy, D8 Projection, D9 Rationalization, D10 Denial, D11 Omnipotence, D12 Idealization, D13 Devaluation, D14 Repression, D15 Dissociation, 16 Reaction formation, D17 Displacement, D18 Undoing, D19 Intellectualization, D20 Isolation of affect, D21 Affiliation, D22 Altruism, D23 Anticipation, D24 Humor, D25 Self-assertion, D26 Self-observation, D27 Sublimation, and D28 Suppression.

The network structure of defense mechanisms is displayed in Figure 1. Visual inspection revealed a main component including 16 nodes: *Passive aggression* (D3), *Splitting of other's image* (D4), *Splitting of self-image* (D5), *Projective identification* (D6), *Autistic fantasy* (D7), *Projection* (D8), *Denial* (D10), *Devaluation* (D13), *Repression* (D14), *Dissociation* (D15), *Reaction formation* (D16), *Altruism* (D22), *Anticipation* (D23), *Self-assertion* (D25), *Self-observation* (D26), and *Suppression* (D28). In contrast, the remaining nodes showed only one or no connection with other variables of the network. The correlation matrix and the centrality indices are shown in Supplementary Figure S1 and Figure 2, respectively. The highest node strength was observed for *Self-assertion* (D25), which was positively connected to *Altruism* (D22), *Self-observation* (D26), and *Suppression* (D28) and negatively connected to *Passive aggression* (D3), *Splitting of self-image* (D5), *Autistic fantasy* (D7), *Projection* (D8), *Devaluation* (D13), and *Dissociation* (D15). Among immature defense mechanisms (clusters 1–4 of the DMRS-SR-30), the defense with the highest node strength was *Passive aggression* (D3), which was positively connected to *Splitting of other's image* (D4) and *Projective identification* (D6) and negatively correlated to *Altruism* (D22), *Anticipation* (D23), *Self-assertion* (D25), *Self-observation* (D26), and *Suppression* (D28). The strongest connection was between *Intellectualization* (D19) and *Isolation of affect* (D20). *Acting out* (D1, a defense of the most immature cluster) was isolated from the main component of the network, showing a positive connection only with *Help-rejecting complaining* (D2; another defense of the most immature cluster). Several defense mechanisms did not exhibit significant connections with other variables of the network, including *Affiliation* (D21), *Humor* (D24), *Sublimation* (D27) (i.e., three mature defenses) *Undoing* (D18), *Displacement* (D17), and *Rationalization* (D9). Among the 16 defenses included in the main component, *Autistic fantasy* (D7), *Projection* (D8), *Denial* (D10), and *Reaction formation* (D16) displayed only weak connections with other nodes. Overall, defenses belonging to the same clusters of the DMRS-SR-30 played different roles in the network—for example, *Self-Assertion* (D25; a mature defense) was the most central node of the network, while *Sublimation* (D27; another mature defense) did not show any significant connection with any other variable; likewise *Acting out* (D1; an immature defense of the action level) and *Help-rejecting complaining* (D2; another defense of the action level) were strongly related to each other but remained isolated from *Passive aggression* (D3; the third defense of the action level), which was instead included in the network. CS was 0.35 for Strength (Supplementary Figure S2). The bootstrapped confidence intervals of the estimated edge weights are displayed in the Supplementary Figure S3.

### Associations between defenses and symptoms

To further understand results of the network analysis, we tested associations between defenses and symptoms of depression and anxiety. Table 2 shows Pearson correlations between all DMRS-SR-30 subscales and the two SCL-90 subscales for depression and anxiety. As expected, ODF and mature defenses (Factor 1) were negatively related to symptoms (*r* ranging from −0.17 to −0.29; all *p* < 0.01), while immature defenses (Factor 3) were positively related to both depression (*r* = 0.34*; p* < 0.01) and anxiety (*r* = 0.17*; p* < 0.01). Mental inhibition and avoidance defenses (Factor 2) was instead related to depression but unrelated to anxiety symptoms.

**Table 2:** Correlations between defenses and symptoms of depression and anxiety.

	**Depression (SCL-90-DEP)**	**Anxiety (SCL-90-ANX)**
ODF	**−0.29** ^**^	**−0.17** ^**^
**DMRS-SR-30 FACTORS**		
Factor 1: Mature	**−0.24** ^**^	**−0.17** ^**^
Factor 2: Mental inhibition and avoidance	**−0.09** ^*^	0.03
Factor 3: Immature depressive	**0.34** ^**^	**0.17** ^**^
**Defense levels**		
L1: Action defense level	**0.14** ^**^	**0.12** ^**^
L2: Major image distorting defense level	**0.33** ^**^	**0.13** ^**^
L3: Disavowal defense level	0.03	0.03
L4: Minor image distorting defense level	**0.15** ^**^	0.05
L5: Neurotic defense level	−0.04	0.03
L6: Obsessional defense level	−0.05	0.03
L7: High-adaptive defense level	**−0.24** ^**^	**−0.17** ^**^
**Individual defenses**		
D1: Acting out	0.07	0.06
D2: Help-rejecting complaining	**0.15** ^**^	**0.12** ^**^
D3: Passive aggression	**0.16** ^**^	0.05
D4: Splitting of object's image	**0.12** ^**^	**0.08** ^*^
D5: Splitting of self-image	**0.36** ^**^	**0.10** ^*^
D6: Projective identification	**0.13** ^**^	0.07
D7: Autistic fantasy	**0.12** ^**^	**0.10** ^*^
D8: Projection	**0.10** ^*^	0.03
D9: Rationalization	**0.08** ^*^	**0.08** ^*^
D10: Denial	**−0.22** ^**^	**−0.14** ^**^
D11: Omnipotence	**−0.14** ^**^	**−0.08** ^*^
D12: Idealization	0.02	0.00
D13: Devaluation	**0.32** ^**^	**0.12** ^**^
D14: Repression	−0.02	−0.03
D15: Dissociation	**0.17** ^**^	**0.17** ^**^
D16: Reaction formation	−0.04	−0.02
D17: Displacement	**−0.16** ^**^	−0.02
D18: Undoing	−0.01	0.05
D19: Intellectualization	−0.05	0.01
D20: Isolation of affects	−0.04	0.01
D21: Affiliation	0.01	**0.10** ^*^
D22: Altruism	**−0.16** ^**^	**−0.10** ^*^
D23: Anticipation	0.02	−0.05
D24: Humor	**−0.17** ^**^	**−0.18** ^**^
D25: Self-assertion	**−0.26** ^**^	**−0.17** ^**^
D26: Self-observation	−0.01	**−0.09** ^*^
D27: Sublimation	**−0.17** ^**^	**−0.10** ^*^
D28: Suppression	**−0.20** ^**^	−0.07

The DMRS-SR-30 factorial structure has been documented in Prout et al. (2022). ^**^p < 0.01; ^*^ *p* < 0.05. The bold values correspond to the significant correlation coefficients.

Among mature defenses, *self-assertion* (a central node in the network), *humor, sublimation*, and *altruism* were negatively associated with both symptoms. Moreover, *suppression* was also negatively related to depression, while *affiliation* and *self-observation* were negatively related to anxiety. Conversely, *passive aggression*, which resulted as another central node in the network, was positively associated with depression (*r* = 0.16*; p* < 0.01), together with most of the depressive defenses (i.e., *help-rejecting complaining, splitting of self and object's image, projective identification, projection*, and *devaluation*) and some other immature defenses (*rationalization* and *autistic fantasy*). Positive correlations between immature defenses and anxiety resulted in lower intensity and included only *help-rejecting complaining, splitting of self and object's image, rationalization, autistic fantasy*, and *devaluation*. Interestingly, the immature defenses *denial* (*r* ranging from −0.14 to −0.22; all *p* < 0.01) and *omnipotence* (*r* ranging from −0.08 to −0.14; *p* ranging from < 0.05 to < 0.01) and the neurotic defense *dissociation* (*r* = 0.17*; p* < 0.01) showed negative and positive association with symptoms, respectively. Finally, *displacement* was found negatively related to depression, while *affiliation* was found positively related to anxiety.

## Discussion

### The network structure

The present study encompasses the innovative representation of defense mechanisms as active components in a complex system (Borsboom et al., 2021) that mutually influence each other (Roefs et al., 2022) giving rise to the “emerging property” of defensive functioning.

Visual inspection of the network structure showed a main interacting component that includes defense mechanisms of different levels: mature, neurotic and immature defenses. This implies that the interaction between different defense mechanisms is not directly dependent on their level of maturity; conversely defenses characterized by the same level of maturity can play a very different role in the network. According to the network approach, defense mechanisms of the same cluster play different roles in the network structure. For example, *humor* and *self-assertion*, both mature defenses, play very different roles in the network: while *self-assertion* can affect several other defenses functioning through its connections, *humor* has no effect at all, resulting an isolated node. Similarly, *passive aggression* shows a relevant impact, but does not appear closely connected to the other defenses of the action defense level. In fact, the *acting out* mechanism was isolated, showing only a positive connection with *help-rejecting complaining*.

Centrality indices reveal that *self-assertion* is the most central node in the whole network, whereas *passive aggression* is the most central node while considering only immature defenses.

In our network structure, *self-assertion* showed positive connections with *suppression, altruism* and *self-observation*, and negative connections with *passive aggression, dissociation* and *splitting of self-image*. Individuals employing *self-assertion* deal “with emotional conflicts, or internal or external stressors, by expressing one's feelings and thoughts directly to achieve goal” (Di Giuseppe and Perry, 2021). Thus, based on our result, we may hypothesize that *passive aggression, dissociation* and *splitting of self-image* limit the capacity to be assertive in dealing with internal and external conflicts. Interestingly, while *self-assertion* is not manipulative or coercive, the individual employing passive aggression is resentful, hostile and expresses his feelings in an un-assertive way (Di Giuseppe and Perry, 2021). Notably, the use of *self-assertion* might hence be protective in individuals with depressive and anxiety symptoms because it allows the person to function without the anxiety or guilt associated with unexpressed emotions (Perry and Bond, 2017; Martino et al., 2019; de Roten et al., 2021).

As mentioned earlier, *self-assertion* was positively connected with three high-adaptive defenses. According to the hierarchical model of defense mechanisms and empirical investigations (e.g., Di Giuseppe and Perry, 2021; Perry et al., 2022; Tanzilli et al., 2021), mature defenses are associated with higher awareness, lower psychopathology, and the capability to cope in an optimal way with internal and external stressors.

Regarding immature defenses, *passive aggression* was positively connected with two defenses featuring in the Major Image-Distorting Defense Level: *projective identification* and *splitting of other's image*. According to our network structure, it may be hypothesized that *passive aggression, splitting of other's image* and *projective identification* can reinforce each other and generate a feedback loop in which the individual is “stuck” in an immature defensive stance, hindering the possibility to integrate different views, and to express anger in indirect ways and reacts to non-real (or partially real) threats (Prout et al., 2019; Di Giuseppe and Perry, 2021; Taubner et al., 2023).

Overall, our network structure suggests that, beyond symptom presentation, individuals with depressive and anxiety symptoms exhibit a range of interconnections among defenses which modulate responses to intrapersonal and interpersonal conflicts and stressors. Moreover, mature and immature defenses may play a crucial role in anxiety and depression, as both *self-assertion* and *passive aggression* were highly central in the network structure. Additional details on the role of defenses in the context of symptom presentation are reported in the Supplementary material S4.

### Associations between defenses and symptoms

Two central nodes in the network, namely *self-assertion* and *passive aggression*, were negatively associated with depressive and anxiety symptoms and positively associated with depressive symptoms, respectively. These are crucial findings in light of the opposite (“conflicting”) role they play in the defensive functioning of highly depressed and anxious individuals. On one hand, *passive aggression* is characterized by a facade of overt compliance masking covert resistance toward others. For instance, the subject can fail to express themselves adequately (e.g., being silent for a long while), instead finding indirect and annoying ways to show their opposition to other's influence, which can be associated with depressive symptoms. On the other hand, *self-assertion* deals with emotional conflict through the direct expression of one's feelings or wishes without feeling guilty or ashamed if unsuccessful. An example could be when the subject can disagree with others and express opinions without being overly hostile, devaluing, or manipulative of others.

### Implications

This study provides a network model of defense mechanisms of depression and anxiety. The interconnected nature of our network could resemble the complexity found in cognitive-behavioral models of depression and anxiety, which emphasize the interplay between thoughts, emotions, and behaviors and the impact of cognitive distortions on symptoms (Beck et al., 1979; Powers et al., 2017). Additionally, the Psychodynamic Diagnostic Manual −2nd Edition (Lingiardi and McWilliams, 2017) suggests that there is considerable complexity in the subjective experiences of symptom patterns in anxiety and depression, including affective states, cognitive patterns, somatic states, and relationship patterns.

Recognizing complexity has clinical implications. According to our findings, it could be argued that targeting defenses without relevant connections may not significantly improve defensive functioning. In contrast, psychotherapy targeting highly central defense mechanisms (i.e., *self-assertion* and *passive aggression*, as they play a crucial role in our network) might be a more promising strategy since central nodes display more links with other variables in the network. However, longitudinal studies are needed to test this hypothesis. Moreover, *passive aggression* and *self-assertion* are closely interconnected, suggesting that therapy could benefit from reducing passive aggression while enhancing *self-assertion*. This dual approach would help individuals navigate social contexts more effectively, manage stress, and build resilience, ultimately improving their capacity to adapt and positively influence their environment (Perry, 1990).

This study also advances theoretical and clinical understanding of defense mechanisms. A system where mature defenses influence each other could act as a protective factor, fostering emotion regulation and adaptive interpersonal skills. In contrast, a system where immature defenses influence each other could represent a risk factor, limiting resilience and flexibility.

### Limitations and future research

Our findings should be read in light of the study limitations. One limitation is that participants were recruited during the first wave of the COVID-19 pandemic, a period marked by increased stress and uncertainty (World Health Organization, 2022). This prompted the use of both mature defenses, which played a protective role, and immature defenses, which were associated with mental health symptoms (Di Giuseppe et al., 2021). To corroborate our findings, replication studies conducted in non-pandemic periods are warranted.

Participants were recruited through an online survey, involving an unequal proportion of women and men in our sample (Di Giuseppe et al., 2021). Although this recruitment procedure is increasingly used in research (Altuncu et al., 2023) and typically contemplates this sample imbalance, this may have led to a slight overrepresentation of defense mechanisms more frequently associated with women. For example, *acting out* and *projection*, which are more prevalent among men, may have been underrepresented, while women tend to use more internalizing defenses, such as *repression* (Cramer, 2000). These documented differences in the use of defenses across genders may have impacted the interconnections in the network; however, further research is warranted.

Furthermore, the use of self-reported measures might have biased the self-assessment of psychological variables due to the effect of social desirability. Further research using clinician- or observer-rated instruments on defenses will be needed to test the generalizability of our results. Moreover, the Italian version of the SCL-90 (the measure we adopted) has no gold-standard cut-offs to identify clinically significant depressive and anxiety symptoms. Given this limitation, we adopted the well-established thresholds of the updated version of this measure, the SCL-90-R (showing crucial similarities with the previous version; Derogatis and Cleary, 1977; Prunas et al., 2012), to identify the study participants. Finally, this study was conducted in the general population, warranting a replication study in clinical samples with both depressive and anxiety mental health conditions.

## Conclusion

This is the first study conceptualizing defensive functioning as a complex system. Results of this study might inform future research questions aimed at detecting transdiagnostic intervention targets in individuals with symptoms of depression and anxiety.

## Data Availability

The datasets presented in this article are not readily available because the data that support the findings of this study are available upon reasonable request. Requests to access the datasets should be directed to gabriele.lobuglio@uniroma1.it.
