# Supplementary material for: Defense mechanisms in individuals with depressive and anxiety symptoms: a network analysis
Source: Front Psychol. 2024 Nov 8;15:1465164. doi: 10.3389/fpsyg.2024.1465164 (PMC11581944; doi:10.3389/fpsyg.2024.1465164)
Supplement: Supplementary file 1 [file Table_1.DOCX]

**Index**

**Figure S1.** Correlation matrix

**Figure S2**. Average correlation between centrality indices of network subsamples and the original sample

**Figure S3.** Bootstrapped confidence intervals of estimated edge weights

**S4.** Supplementary discussion.

**Figure S1. Correlation matrix**


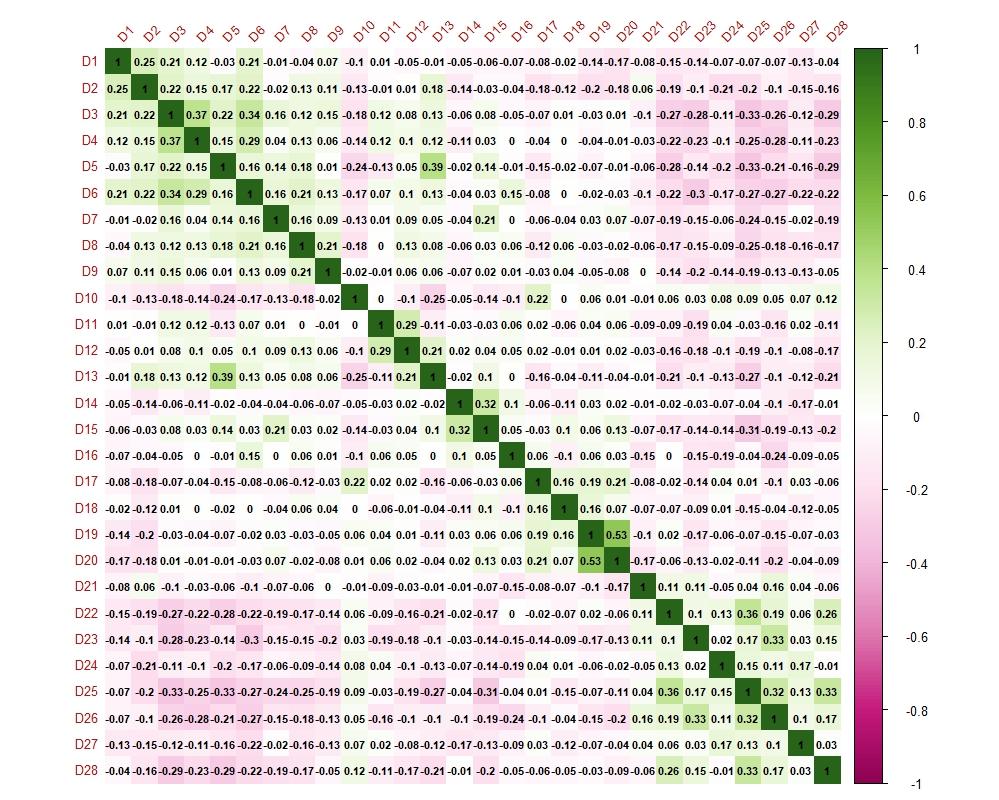


Legend Figure S1: D1 Acting out, D2 Help-rejecting complaining, D3 Passive aggression, D4 Splitting of other's image, D5 Splitting of self-image, D6 Projective identification, D7 Autistic fantasy, D8 Projection, D9 Rationalization, D10 Denial, D11 Omnipotence, D12 Idealization, D13 Devaluation, D14 Repression, D15 Dissociation, 16 Reaction formation, D17 Displacement, D18 Undoing, D19 Intellectualization, D20 Isolation of affect, D21 Affiliation, D22 Altruism, D23 Anticipation, D24 Humor, D25 Self-assertion, D26 Self-observation, D27 Sublimation, D28 Suppression.


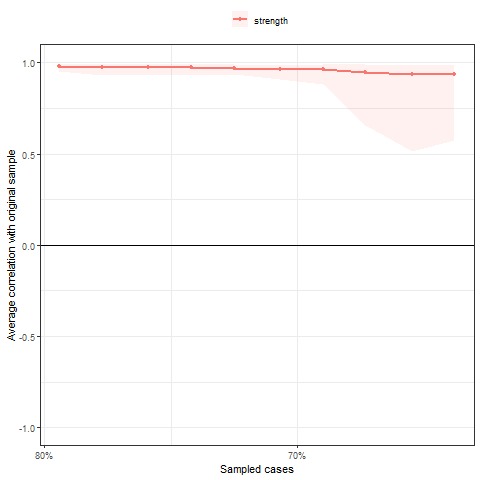


**Figure S2.** Average correlation between centrality indices of network subsamples and the original sample in the network. Lines indicate means and areas indicate the range from the 2.5th quantile to the 97.5th quantile.


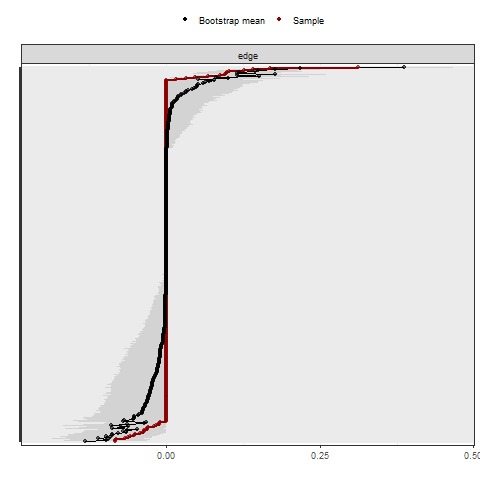


**Figure S3.** Bootstrapped confidence intervals of estimated edge weights.

**Supplementary discussion**

. Our network structure suggests that *self-assertion* is connected with the capability to fulfill the needs of other individuals (altruism), reflect on their own motivation, feelings, behavior and thoughts (*self-observation*) and actively and temporarily not thinking about disturbing problems (*suppression*).

*Passive aggression* was associated with both *splitting of other’s image* and *projective identification*. Individuals using the *splitting of other’s image* view other individuals in terms of all good or all bad and display contradictory feelings and expectations that fail to be reunited into a coherent whole. *Projective identification* is characterized by the projection of unacceptable impulses into other individuals with the attribution of those feelings as a result of the interaction with the other person (who is “depositary” of the projected material).

From a theoretical point of view, *passive aggression* can also be understood as one of the possible ways of exerting interpersonal pressure for the other person to identify with the projected content–– that is, to enact *projective identification*. The interrelation between these defense mechanisms found in our network may suggest that the unrecognized/implicit feelings of anger (acted out by means of *passive aggression* and/or expressed intersubjectively through *projective identification*) may have a crucial role in individuals with symptoms of depression, as recognized in the psychoanalytic literature (Abraham, 1911; Busch, 2009; Freud, 1917; Painuly et al., 2005).

In contrast, the clinical depiction of self-assertion is at odds from commonly reported manifestations of depression (e.g., fatigue, excessive guilt, indecisiveness, feelings of worthlessness) and anxiety (e.g., fear in social contexts, avoidance of stressful situations, and excessive worry) (Perry, 1990).

**Supplementary references**

Abraham, K (1911). Notes on the psycho-analytical investigation and treatment of manic-depressie insanity and allied conditions. In *Selected Papers on Psychoanalysis*, 137–56. Hogarth Press, 1927.

Busch, F. (2009). Anger and depression. *Advances in Psychiatric Treatment, 15*(4), 271-278. doi:10.1192/apt.bp.107.004937

Freud, S. (1917). Mourning and melancholia. In The Standard Edition of the Complete Psychological Works of Sigmund Freud, Volume 14 (ed Strachey, J) 239–58. Hogarth Press, 1953.

Painuly, N., Sharan, P., & Mattoo, S. K. (2005). Relationship of anger and anger attacks with depression: a brief review. European archives of psychiatry and clinical neuroscience, 255(4), 215–222. <https://doi.org/10.1007/s00406-004-0539-5>

Perry J. C. (1990). *Defense Mechanism Rating Scales (DMRS),* 5th Edn. Cambridge, MA: Author.
